# Tree Species-Dependent Inactivation of Coronaviruses and Enteroviruses on Solid Wood Surfaces

**DOI:** 10.1021/acsami.4c02156

**Published:** 2024-05-28

**Authors:** Sailee Shroff, Anni Perämäki, Antti Väisänen, Pertti Pasanen, Krista Grönlund, Ville H. Nissinen, Janne Jänis, Antti Haapala, Varpu Marjomäki

**Affiliations:** † Department of Biological and Environmental Sciences and Nanoscience Center, University of Jyväskylä, Jyväskylä 40500, Finland; ‡ Department of Environmental and Biological Sciences, University of Eastern Finland, Kuopio 70210, Finland; § Department of Chemistry, Sustainable Technologies, University of Eastern Finland, 80100 Joensuu, Finland; ∥ FSCN Research Centre, Mid Sweden University, SE-85170 Sundsvall, Sweden

**Keywords:** antiviral, coronavirus, enterovirus, persistence, solid surface, wood

## Abstract

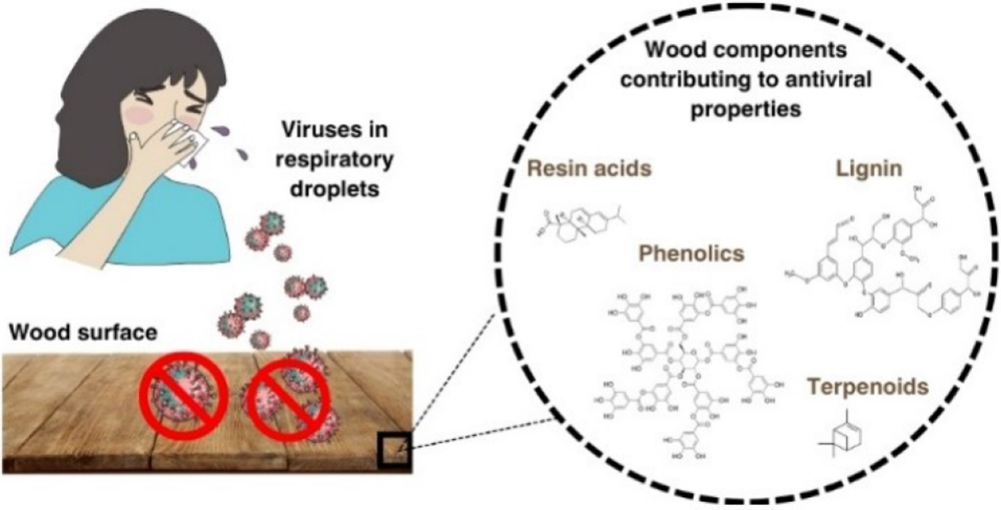

The ongoing challenge of viral transmission, exemplified by the Covid pandemic and recurrent viral outbreaks, necessitates the exploration of sustainable antiviral solutions. This study investigates the underexplored antiviral potential of wooden surfaces. We evaluated the antiviral efficacy of various wood types, including coniferous and deciduous trees, against enveloped coronaviruses and nonenveloped enteroviruses like coxsackie virus A9. Our findings revealed excellent antiviral activity manifesting already within 10 to 15 min in Scots pine and Norway spruce, particularly against enveloped viruses. In contrast, other hardwoods displayed varied efficacy, with oak showing effectiveness against the enterovirus. This antiviral activity was consistently observed across a spectrum of humidity levels (20 to 90 RH%), while the antiviral efficacy manifested itself more rapidly at 37 °C vs 21 °C. Key to our findings is the chemical composition of these woods. Resin acids and terpenes were prevalent in pine and spruce, correlating with their antiviral performance, while oak’s high phenolic content mirrored its efficacy against enterovirus. The pine surface absorbed a higher fraction of the coronavirus in contrast to oak, whereas enteroviruses were not absorbed on those surfaces. Thermal treatment of wood or mixing wood with plastic, such as in wood-plastic composites, strongly compromised the antiviral functionality of wood materials. This study highlights the role of bioactive chemicals in the antiviral action of wood and opens new avenues for employing wood surfaces as a natural and sustainable barrier against viral transmissions.

## 1 Introduction

Since prehistoric times, wood has played an essential role in tools, utilities, and built environment. The 20th century witnessed excessive exploitation of wood that together with rapid industrial advancements provided several alternatives like plastics and metals in interior surfacing and utilities in our built environment. Recent trends, underlined by sustainability concerns and appreciation for wood’s unique aesthetic and haptic properties are reclaiming the use of wood in many daily uses.^1,2^

Parallel to these material trends, the 21st century is marked by emerging health challenges, notably viral outbreaks, such as SARS and COVID-19. Transmission mechanisms for these viruses include not only direct human-to-human contact but also interactions with contaminated surfaces.^3−5^ Viruses do not replicate outside their host cells; however, they are able to persist for a long period of time on different surfaces as fomites.^6^ While enveloped viruses, such as coronaviruses, exhibit quite short surface persistence up to 5 days, nonenveloped viruses on the other hand, shielded by robust protein capsids, can endure for weeks, often resisting standard disinfection techniques. This is due to the presence of a strong protein capsid which is difficult to break down with disinfectants.^7,8^ While disinfectants remain the primary strategy for neutralizing surface pathogens, their efficacy is limited and their continuous use poses environmental, health, and material degradation concerns.^9,10^

The intersection of these trends points to a need for research into antiviral surfaces, and this has sparked a new interest among scientists in reducing the circulating viral load. Wood holds promise as a material capable of mitigating the spread of pathogens due to its intricate composition and architecture. Currently, the few commercially available antiviral surfaces and disinfectants are the only way to reduce the number of pathogens on solid surfaces. Disinfectants usually exhibit limited efficacy, prolonged use raises health concerns for the user, have an adverse environmental impact, carry the potential for pathogen resistance, and destroy the natural microbiota as well.^10^ Additionally, repeated use of disinfectants reduces the longevity of the surface, for example, disinfectants are known to harden plastic and crack rubber.^9^

Historical practices have showcased wood’s inherent antimicrobial properties, evidenced in traditional methods like the use of wooden boards in cheese and wine production.^11^ The underlying antimicrobial mechanisms are believed to arise from wood’s hygroscopic nature, which promotes rapid drying, and the antimicrobial compounds it naturally contains.^12,13^ However, the interplay of various factors, such as wood type, surface condition, and ambient conditions, necessitates more comprehensive research.^14^

While the antibacterial and antifungal properties of wood have been documented across cultures and time periods, the investigation into its antiviral potential has remained relatively unexplored until recent years. In one study, Greatorex and colleagues showed a reduction of more than 4.2 logs of Influenza A viral titer on pine surface after 24 h.^15^ In another study by Chin and colleagues at the beginning of the COVID-19 pandemic suggested that SARS-CoV-2 viral titer could be reduced by four logs post 24 h treatment on a wood surface.^16^ To the best of our knowledge, there is no literature that demonstrates the persistence of enveloped and nonenveloped viruses on different wood species and after short contact times. Also, the effect of environmental factors such as temperature and relative humidity (RH) or modification of wood surface properties on the persistence of viruses on wood has not been comprehensively tested.

This work aimed to bridge the knowledge gap by investigating the antiviral properties of wood surfaces, emphasizing both enveloped and nonenveloped viruses. Through rigorous analysis, we explored the impact of different wood species, treatments, and environmental conditions on viral persistence. Our findings offer insights into the intricate relationship between the organic chemical compounds of wood and their potential to mitigate viral loads.

## 2 Materials and Methods

### 2.1 Cells, Viruses, and Surfaces

We used two cell lines, MRC-5 and A549 cells, which were obtained from the American Type Culture Collection (Manassas, VA, USA). MRC-5 cells are fibroblast-like cells derived from normal lung tissue obtained from a 14-week-old male fetus. While A549 cells are adenocarcinoma human alveolar basal epithelial cells. MRC-5 cells were cultured in Minimum Essential Medium (MEM), while A549 cells were cultured in Dulbecco’s Modified Eagle Medium (DMEM). Both culture media were supplemented with 10% fetal bovine serum (FBS), 1% penicillin–streptomycin, and 1% GlutaMAX, all from Gibco (Paisley, UK). The cultures were maintained in a humidified incubator with 5% CO_2_ at 37 °C.

Beta Coronavirus 1 (OC43 strain; ATCCVR-1558)(Manassas, VA, USA) was propagated following the protocol by Dent and Neuman with minor modifications while coxsackie virus A9 (CVA9; Griggs strain; ATCC) was produced and purified as described previously by Myllynen et al.^17,18^

Six different varieties of wood species were used in this study: Scots pine (*Pinus sylvestris*), silver birch (*Betula pendula*), gray alder (*Alnus incana*), eucalyptus (*Eucalyptus globulus*), pedunculate oak (*Quercus robur*), and Norway spruce (*Picea abies*). In addition, different coarseness of pine (coarseness: 80, 120, 320, 1000, and planned grit sanded surfaces), wood-plastic composite (about 50% wood flour, 50% polypropylene), and two differently thermo-treated spruce (*Picea abies*) and pine samples (ThermoWood S and D modified timber, see e.g., Cai et al.) were also tested.^19^ Industrial-grade polyethylene (PE) was used as a plastic control sample. The wood materials were sterilized by γ-radiation and the plastic was sterilized with 70% ethanol.

### 2.2 Surface Persistence Studies

For the persistence studies, test samples (wood and plastic surfaces) were placed in 12-well plates. A 5 μL droplet of the virus (corresponding to 8 × 10^4^ PFU) was inoculated onto the center of each sample’s surface. These plates were then transferred to a custom-built humidity chamber (Kenttäviiva Ltd., Finland) for specified time intervals. The chamber’s temperature and relative humidity were adjusted as needed for the experiments (e.g., 21/37 °C and 20/40/60% RH). To achieve higher RH conditions (>90% RH), 6 mL of ddH_2_O was added to each 12-well plate, which was then sealed with parafilm during incubation. After the specified time points, the samples were gently flushed with 995 μL of media and rocked for 1 min to release the virus particles into the medium. The flushed media was collected into Eppendorf tubes placed on ice and further diluted 100 times with media. A 2% MEM solution was used as the flushing media for coronaviruses, while a 1% DMEM solution was used for enteroviruses.

### 2.3 Cytopathic Effect (CPE)

To determine the infectivity of the flushed viruses, we employed the cytopathogenic effect (CPE) inhibition assay. For coronaviruses, MRC-5 cells were cultured for 24 h at 37 °C in 96-well flat-bottomed microtiter plates (Sarstedt, Numbrecht, Germany) at a density of 15,000 cells/well in 100 μL of 10% MEM. Meanwhile, for enteroviruses, A549 cells were cultured under similar conditions with 10% DMEM at a density of 12,000 cells/well.

Following the surface persistence studies, 100 μL of the media flushed from the surfaces and virus control were added to the cultured cells. The plates were then incubated for 5 days at 34 °C for coronaviruses and 2 days at 37 °C for enteroviruses, or until the cytopathic effect was visible. Once the cytopathic effect was observed, the cells were washed twice with PBS and then stained for 10 min using a CPE dye solution (comprising 0.03% crystal violet, 2% ethanol, and 36.5% formaldehyde). Post this, the excess stain was removed with two washes of ddH_2_O. Stained cells were lysed using a CPE lysis buffer (0.8979 g of sodium citrate and 1 N HCl in 47.5% ethanol). The absorbance from the plates was subsequently read at 570 nm using the VICTORTM X4 multilabel reader from PerkinElmer (Turku, Finland).

### 2.4 Statistical Analysis

The results from the CPE assay were plotted as a column graph of the cell viability with standard error means using GraphPad Prism 9 (GraphPad Software, San Diego, CA, USA). The statistical significance was calculated using the one-way analysis of variance (ANOVA), followed by the Bonferroni test (*p < 0.05, **p < 0.01, ***p < 0.001, and ****p < 0.0001).

### 2.5 Direct Flushing

To assess the potential differential absorption of HCoV-OC43 and CVA9 within different wood surfaces, a direct flushing experiment was devised. A 5 μL droplet containing HCoV-OC43 (8 × 10^4^ PFU) and CVA9 (1 × 10^5^ PFU) was applied to the surfaces of pine and oak, followed by a 15 min incubation at room temperature under high RH conditions (>90% RH). Subsequently, the surfaces bearing HCoV-OC43 and CVA9 were flushed with 995 μL of 2% MEM and 1% DMEM, respectively. The quantification of viral RNA in the flush samples was performed using RT-PCR and qPCR methods.

### 2.6 RT-PCR and qPCR

The protocol for the cDNA synthesis using RT-PCR and amplification using qPCR was performed as described previously by Turkki et al.^20^ The reverse primer specific to HCoV-OC43 was 5′-AATGTAAAGATGRCCGCGTATT, and the corresponding forward primer was 5′-TGTTAGGCCRATAATTGAGGAC (Merck). For enterovirus, the reverse primer was 5′-GAAACACGGACACCCAAAGTA, and the forward primer 5′-CGGCCCCTGAATGCGGCTAA. qPCR was used to determine the relative amounts of viral RNA or virus infection between samples in two ways. To evaluate the effects of virus infection, samples were taken from 3-day cultivation from MRC-5 cells after the flushed samples were applied to cells. In contrast, in evaluating the relative amount of viruses that were absorbed on the surfaces, qPCR was performed directly from the flushed samples, without cultivation on cells.

### 2.7 Immunolabeling and Confocal Microscopy

Confocal microscopy was employed to examine the various stages of the coronavirus infection cycle. In this study, two separate experiments were conducted: one with a total infection time of 2 h, and the other with a total infection time of 15 h. At first, a 5 μL droplet of purified HCoV-OC43 virus (2 × 10^6^ PFU) was applied to the Pine and PE surfaces and incubated for 1 h at room temperature with approximately 92% RH. After incubation, the viruses were flushed from the surfaces and added into two 96-well plates containing subconfluent MRC-5 cells at a multiplicity of infection (MOI) of 100. The virus in the 96-well plates was allowed to settle on the host cell surface for 1 h at room temperature. Following this, the plates were transferred to 34 °C for another 1 h. At the end of this incubation period, the cells were gently flushed with PBS to remove any unbound virus. In the plate where the infection continued for the remaining 13 h, the PBS was replaced with 2% MEM and cells were incubated at 34 °C. At the end of the 2 and 15 h incubation period, the cells were fixed, permeabilized, and immunolabeled as per the details mentioned in the Supporting Information. Montages of the images were generated by using Fiji2 software (ImageJ).

### 2.8 Volatile Organic Compounds from Wood Specimen

In order to study easily evaporated chemical components from wood specimens that can interact with virus deposits on the surface, (total) volatile organic compounds ((T)VOCs) emitted by wood specimens (25 × 25 × 10 mm in dimensions) at fixed 25 and 40 °C temperatures were collected in two consecutive tests using Tenax TA adsorbent tubes (Markes International Inc., Sacramento, CA) containing 200 mg of sorbent (Figure S1A). The first test employed untreated, dry wood specimens. The specimens were dampened before the second test by submerging them in water for one h and sealing them in airtight zip bags for 24 h. The specimens were left to sit for two h under normal laboratory conditions before the second set of VOC sampling after the 24 h of moisture absorption to get rid of excess surface moisture. Hence a total of four samples were collected from each wood species. Sample collection adopts features from the Finnish Building Information Foundation’s (2023) method to evaluate and classify chemical emissions from building materials and the ISO 16000–6:2021 standard (International Organization for Standardization 2021) for active VOC sampling. Further details on the analysis method are given in the supplementary document.

### 2.9 Chemical Fingerprinting of Semivolatile Chemicals from Wood Specimen

To further characterize and classify the wood specimen’s chemical composition, semivolatile organic compounds (SVOCs) were determined by using thermal desorption connected directly to the high-resolution mass spectrometer. Experiments were performed on a Bruker timsTOF quadrupole time-of-flight (Q-TOF) instrument (Bruker Daltonics GmbH, Bremen, Germany), equipped with a direct insertion probe (DIP) fitted into an atmospheric-pressure chemical ionization (APCI) source (Figure S2A). The DIP device allows direct analysis of semivolatile (polar and nonpolar) compounds without the need for sample preparation, which well complements the conventional VOC analysis using TD-GC-MS. Further details on the analysis method are given in the supplementary document.

The instrument was operated, and the data were acquired using Bruker qtofControl 2.1 software, and the data postprocessing was accomplished by Bruker DataAnalysis 5.2 software. The van Krevelen (VK) diagrams (i.e., a plot of atomic H/C to O/C ratio for each detected compound) were made using CERES Viewer 1.8 software. The compound classifications were based on the criteria proposed elsewhere.^21^ Some compounds were further identified using the CompoundCrawler database search engine.

## 3 Results

### 3.1 Persistence of Human Coronavirus on Different Wood Species

We used the CPE assay to determine the persistence of HCoV-OC43 on various wood species. The tested wood species included Scots pine, silver birch, gray alder, eucalyptus, pedunculate oak, and Norway spruce. We examined both shorter (1–15 min) and longer (1–4 h) incubation times of the virus with the wood surface. The results obtained from the CPE assay highlighted significant differences in the persistence of HCoV-OC43 on different wood surfaces, especially in shorter time intervals.

**Figure 1:**
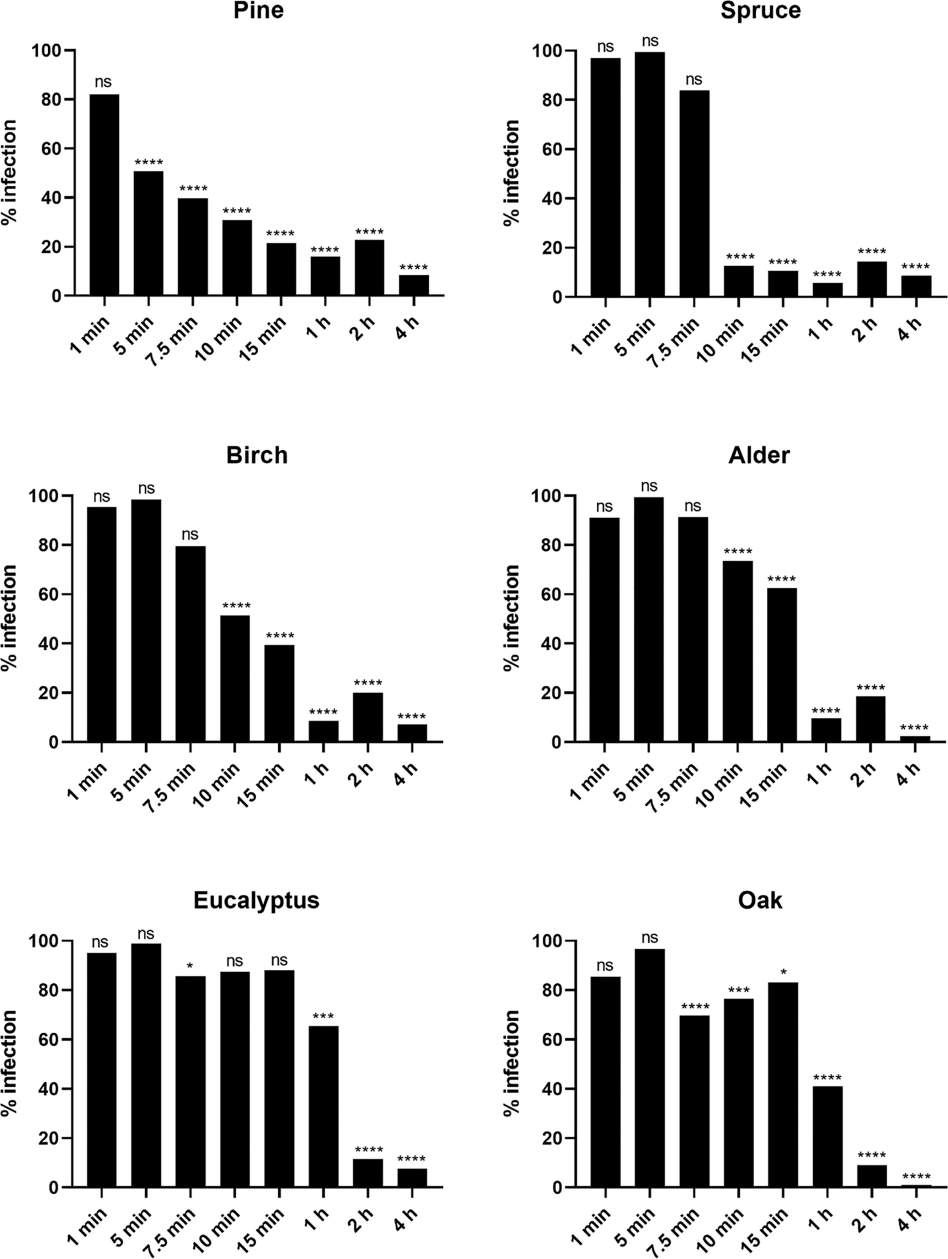
Infectivity of HCoV-OC43 recovered from six different wood species after varying incubation times at room temperature and high RH (>90%). The results have been normalized against the control virus infection which was set to 100% and against the cell control which was set to 0%. Virus without any surface treatment was used as a virus control (VC). All the results are presented as an average + standard errors of the mean (SEM). The statistically significant differences between the test samples and VC are indicated with asterisks: *p < 0.05, ***p < 0.001, ****p < 0.0001, ns is not significant (analyzed with one-way ANOVA with Bonferroni test).

Notably, on the pine surface, the viral infectivity started to reduce as early as after 5 min of incubation on the surface (Figure 1), while on the spruce surface, the infectivity declined drastically starting after 10 min. In the case of birch and alder surfaces, virus infectivity decreased within the tested shorter incubation times but did not reach as high effectivity as pine and spruce. Strikingly, eucalyptus and oak surfaces could not reduce the infectivity of the HCoV-OC43 virus within the shorter incubation times.

When we extended the incubation times of the virus on the surface up to 1 h, pine, spruce, birch, and alder exhibited full reduction of infectivity, while eucalyptus and oak exhibited full activity only after 2 h (Figure 1). These results indicated that eucalyptus and oak were not as expeditious as other wood species in inactivating HCoV-OC43 on its surface and they could be potential fomites to transmit coronaviruses during the first few hours.

### 3.2 Effect of Relative Humidity and Temperature on Persistence on HCoV-OC43 on Different Wood Species

Initial assessments on wood surfaces were conducted under conditions of elevated humidity (exceeding 90% RH) at room temperature in order to maintain optimal conditions for virus infectivity (Figure 1). However, recognizing that real-world conditions vary considerably, it was imperative to examine how fluctuations in temperature and humidity might influence our observations. To this end, experiments were orchestrated across two distinct temperatures, 21 °C (reflective of Nordic indoor environments) and 37 °C (reminiscent of tropical climates). Furthermore, we selected three RH levels: 20, 40, and 60%, thereby encompassing a spectrum ranging from 20 to 90%, which aligns with the range frequently encountered both indoors and outdoors, irrespective of seasonal variations.

**Figure 2:**
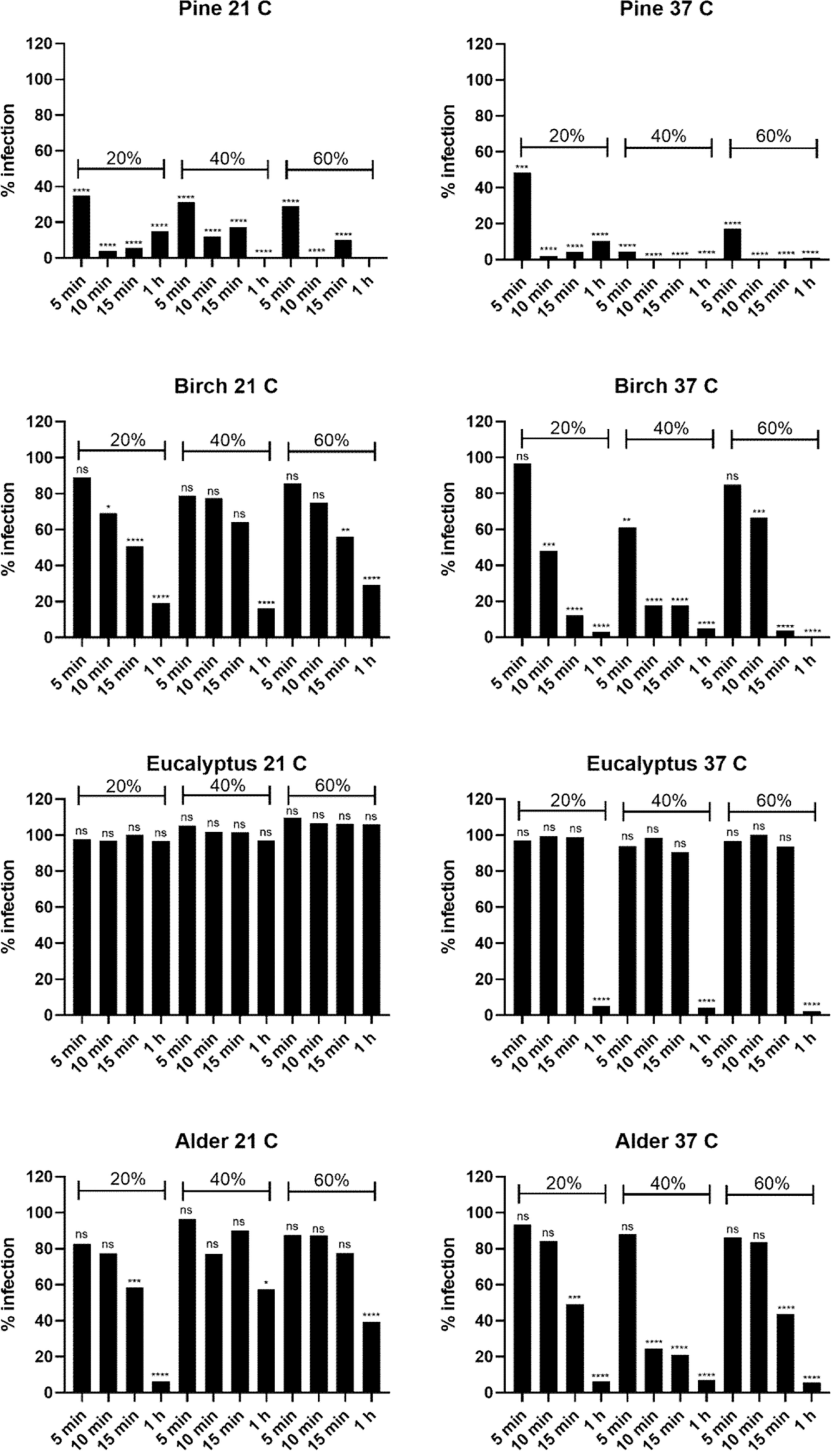
Infectivity of HCoV-OC43 recovered from pine, birch, alder, and eucalyptus surfaces after incubating for various time periods (5 min, 10 min, 15 min, and 1 h) at 21 and 37 °C under different RHs (20, 40, and 60%). The results have been normalized against the control virus infection which was set to 100% and against the cell control which was set to 0%. Virus (VC) without any surface treatment was used as a positive control. All the results are presented as an average + standard errors of the mean (SEM). The statistically significant differences between the test samples and VC are indicated with asterisks: *p < 0.05, **p < 0.01, ****p < 0.0001, ns is not significant (analyzed with one-way ANOVA with Bonferroni test).

Comparative analyses between the two temperature settings revealed that viral infectivity was markedly reduced at 37 °C relative to that at 21 °C (Figure 2). Eucalyptus wood offered a clear illustration of this trend. Here, the virus was entirely inactivated post 1 h of exposure at 37 °C. However, the viral entities persisted and remained infectious under similar conditions at 21 °C. Similarly, the silver birch surface demonstrated total viral inactivation within a mere 15 min at 37 °C, in contrast to a more moderate effect at 21 °C. This thermal effect aligns with extant literature. For instance, Wang et al. delineated how reduced temperatures, coupled with heightened humidity, often prolong the survivability of coronaviruses on surfaces.^22^

In contrast to the influence of the temperature, there appears to be no direct linear relationship between different humidity levels and the loss of virus infectivity. At 21 °C, variations in humidity, from low to high, appeared inconsequential in terms of influencing the virus infectivity. However, at higher temperatures, extreme RHs, such as 20 and 90%, supported the virus’s persistence for a longer duration compared to 40% (Figure 2). A salient observation emerged when assessing virus behavior on alder wood at 37 °C. While at both 20 and 90% RH, the virus was rendered noninfectious after a 15 min exposure, a swifter inactivation occurred at 40% humidity, where the virus lost its infectivity in under 10 min. These results, however, altogether suggest that RH plays a minor role in the inactivation of viruses, whereas temperature plays a bigger role.

### 3.3 Delayed Inactivation of Nonenveloped Viruses on Different Wood Surfaces

**Figure 3:**
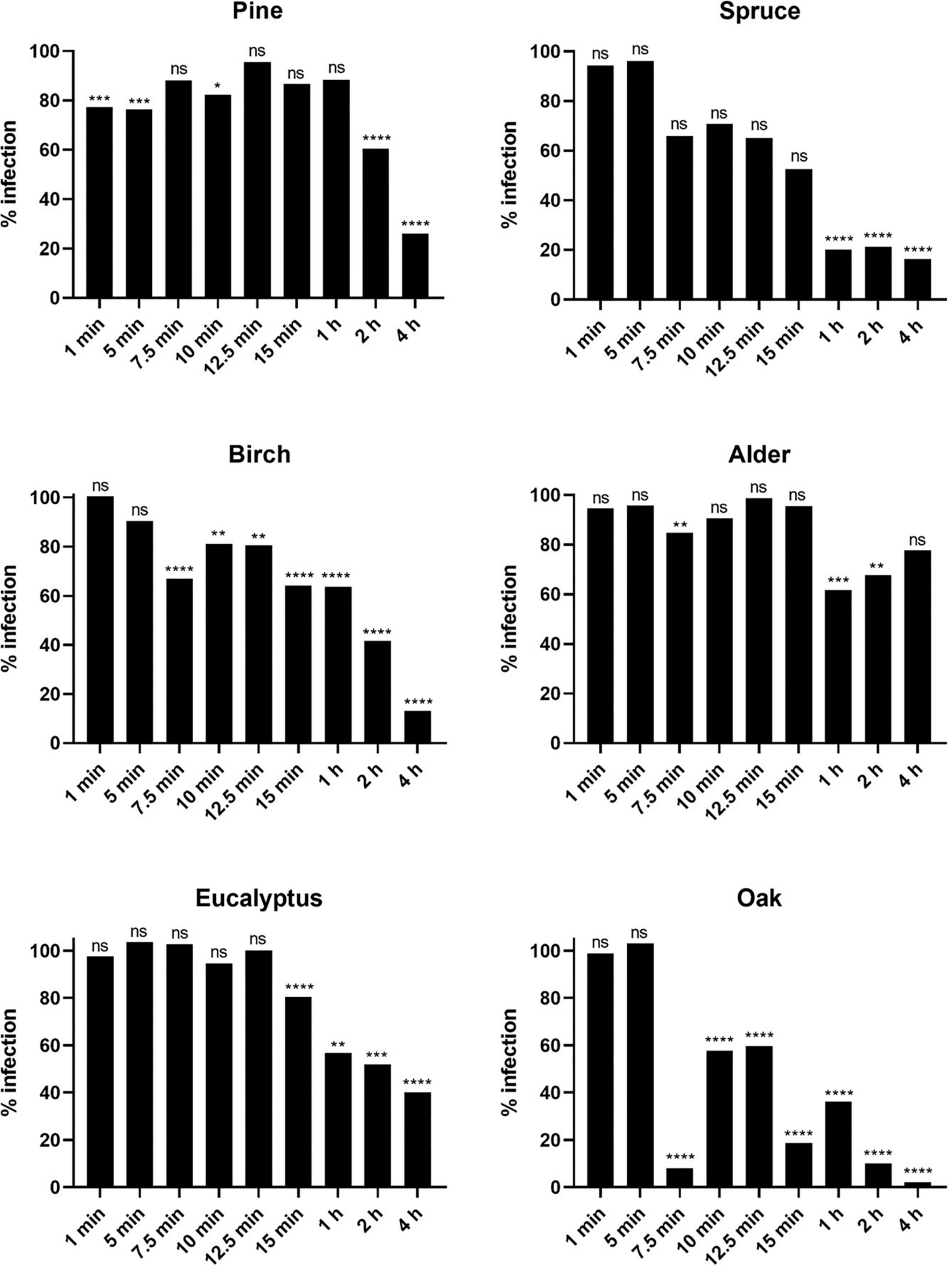
Infectivity of CVA9 recovered from six different wood species after varying incubation time periods at room temperature and high RH (>90%). The results have been normalized against the control virus infection which was set to 100% and against the cell control which was set to 0%. Virus (VC) without any surface treatment was used as a positive control. All the results are presented as an average + standard errors of the mean (SEM). The statistically significant differences between the test samples and VC are indicated with asterisks: *p < 0.05, **p < 0.01, ***p < 0.001, ****p < 0.0001, ns is not significant (analyzed with one-way ANOVA with Bonferroni test).

In addition to the enveloped viruses, it was equally important to also test more stable nonenveloped viruses like CVA9 on the same surfaces. The persistence of CVA9 was also tested for a shorter time ranging from 1 to 15 min and for longer periods ranging from 1 to 4 h (Figure 3). The results with the shorter time of incubation on the surface showed no significant loss of infectivity on any of the tested wood surfaces except for oak, which showed a loss in viral infectivity already starting at 7.5 min (Figure 3). The results with the longer time of incubation on the surface showed varying results for the different wood species. The virus on the spruce surface showed a complete loss of infectivity after 1 h of incubation, while pine, birch, and eucalyptus showed a good loss of infectivity only after 4 h. Alder showed negligible effect even after long incubation periods. These results demonstrate that specific wood species can affect the persistence of nonenveloped viruses on its surface, but these species are interestingly different from those affecting the enveloped coronaviruses. Oakwood showed the fastest inactivation capability followed by pine, birch, and eucalyptus, while some surfaces like alder showed no antiviral effect.

### 3.4 Coronaviruses and Enteroviruses Differ in Their Absorption to Wood Surfaces

**Figure 4:**
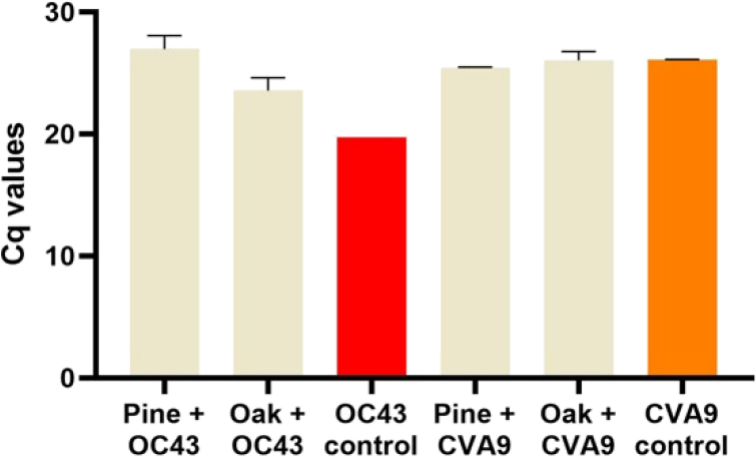
Detection of viral RNA after flushing directly from the (A) pine and (B) oak surface. HCoV-O43 and CVA9 was incubated on the different wood surfaces for 15 min after which the viral RNA in the flush was quantified using RT-PCR and qPCR techniques. In the virus control (VC) sample, the virus has not been incubated on any surface and the input virus has directly been quantified using PCR. All the results are presented as an average plus standard error of the mean (SEM).

In order to decipher the mechanism by which the wood surface demonstrates the antiviral effect, the RNA of the viruses flushed from the wood surface was quantified using qPCR. In addition to pine, oak was chosen as these surfaces had such differing abilities to inactivate coronaviruses and enteroviruses. The viral RNA flushed from the surfaces after a 15 min incubation was first converted to a more stable form of cDNA and then the cDNA was amplified using qPCR. The results from the qPCR revealed that both pine and oak absorbed coronaviruses on the surfaces as their relative amount was clearly lower than the amount of input virus, respectively (Figure 4). A difference of 2.5 and 1.1 logs corresponds to 99.68 and 92.37% reductions in viral RNA on the pine and oak surfaces, respectively, compared to the input virus. Interestingly, enteroviruses showed no difference between the pine and oak surfaces and showed that the virus load was totally flushed away from the surface after 15 min incubation. This is interesting, as oak showed great efficacy against enteroviruses already after 15 min (Figure 3). These results indicate that pine wood absorbs more coronaviruses but not nonenveloped enteroviruses.

### 3.5 Coronaviruses Flushed from the Pine Surface Lose Their Ability To Initiate Infection

**Figure 5:**
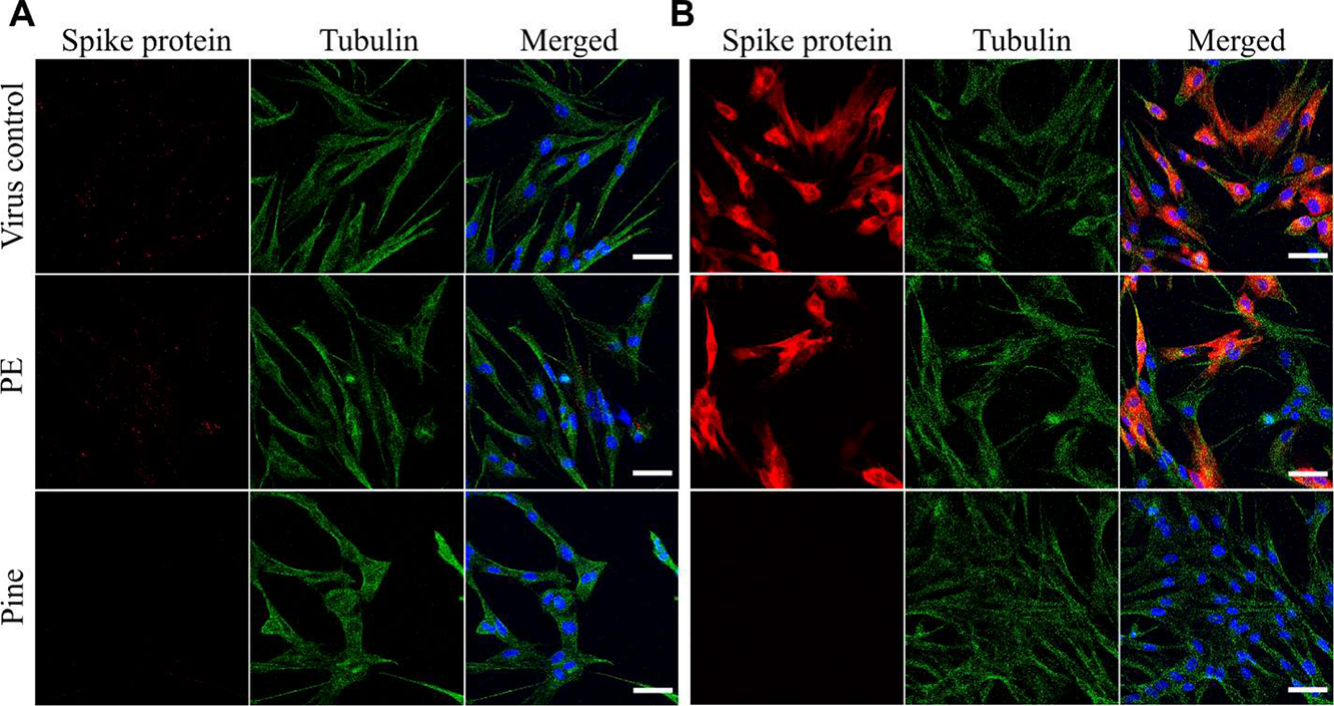
Confocal images of MRC-5 cells infected with HCoV-OC43 flushed from pine and PE surfaces. Time when images were acquired is (A) 2 h and (B) 15 h post infection. The presence of viral spike protein is visible in red, nucleus in blue, and cytoskeletal tubulin in green color. Scale bar: 30 μm.

We tested the infection potential from samples flushed from the pine surface after 1 h treatment by confocal microscopy. In order to follow the infection cycle of coronaviruses inside MRC-5 cells, the spike protein of the virion was labeled and imaged. Two time points were followed in cells: the virus was allowed to proceed with infection for up to 2 h, while in the other case, it was allowed to proceed for 15 h. A virus without surface treatment and viruses flushed from the PE surface were used as controls for comparison. The results from the confocal images after 2 h of infection showed bright red spots within the cellular boundaries in the case of the virus control and viruses flushed from the PE surface, indicating that virus attachment and internalization were initiated in these two cases (Figure 5A). In the case of the viruses flushed from the pine surface, no such observation was made. Further, after 15 h of incubation inside the cells, the viruses flushed from the pine surface did not show cells full of newly synthesized spike proteins like the other samples. This confirmed that the viruses flushed from the pine surface had lost their infection potential (Figure 5B).

### 3.6 Wood Species Have Significantly Different Organic Chemical Compositions

**Figure 6:**
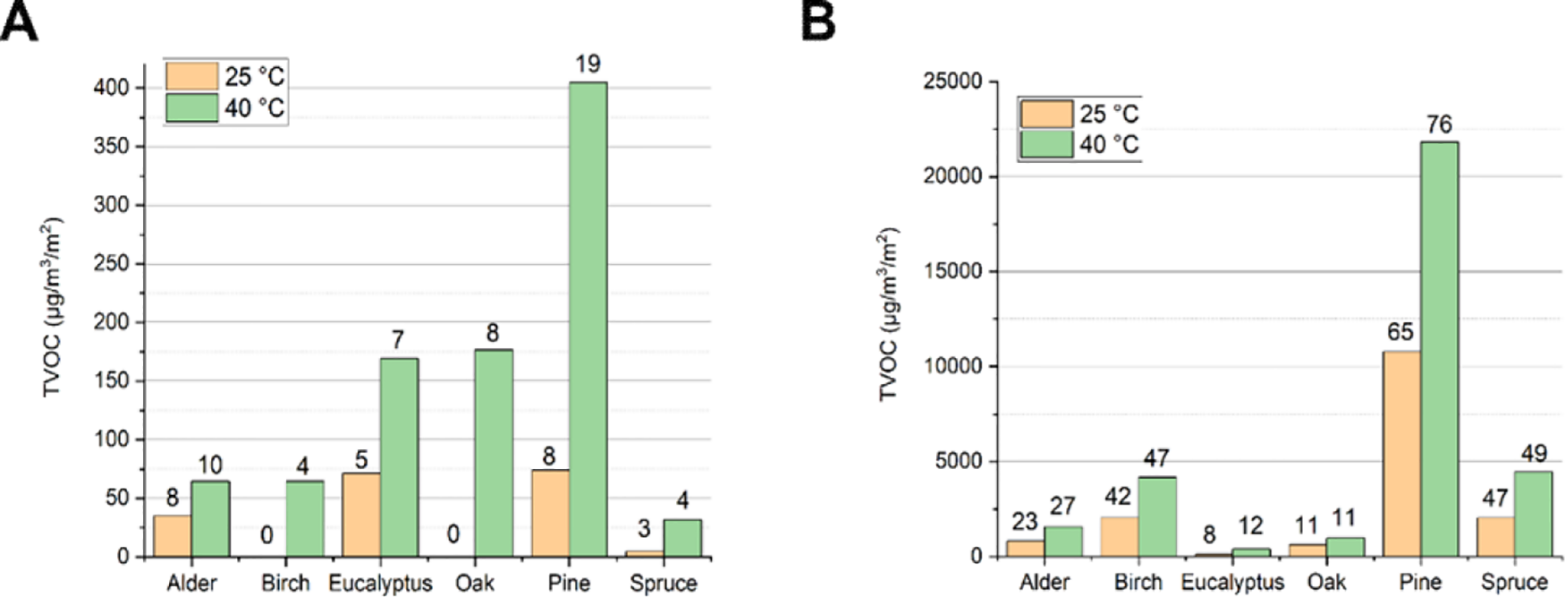
Total volatile organic compound (TVOC) emissions for (A) dry wood specimen and (B) wetted wood specimen, both averages of two samples analyzed. The bars reflect the total emission from wood in 25 and 40 °C conditions while the numbers on top of the bar indicate how many chemical components were identified from each specimen. More detailed results on the 20 most abundant chemicals and total emissions from specimens are provided in Supporting Information Tables S1 and S2.

Wood samples exhibited notable variations in the emission of total volatile organic compounds (TVOCs). To investigate the dissimilarities in chemical composition, particularly regarding easily evaporable substances, emissions were collected and analyzed from both dry and wet wood specimens. The findings revealed a distinct correlation between the emitted chemical compounds at temperatures of 25 and 40 °C and the samples' ability to inactivate viruses. Of the species assessed, Scots pine stood out by registering the highest cumulative emission rate, accompanied by an expansive range of identified chemical entities across both dry and wetted samples (see Figure 6A, B). Similarly, silver birch and spruce posted elevated emission figures, predominantly evident in the wetted samples (as depicted in Figure 6B). Contrarily, the alder, eucalyptus, and oak samples marked significantly subdued emission figures across the board.

Dry wood samples predominantly emitted aldehydes, alcohols, organic acids, and terpenes, although the overall number of components and their volume were relatively low. Conversely, wetted wood specimens consistently showcased more diverse and abundant chemical emissions with the test temperature also exerting a notable influence. The identified components were very similar to those found in dry wood, including terpenes (in pine), aldehydes, alcohols, and organic acids, as well as some cyclic hydrocarbons and ketones.

**Figure 7:**
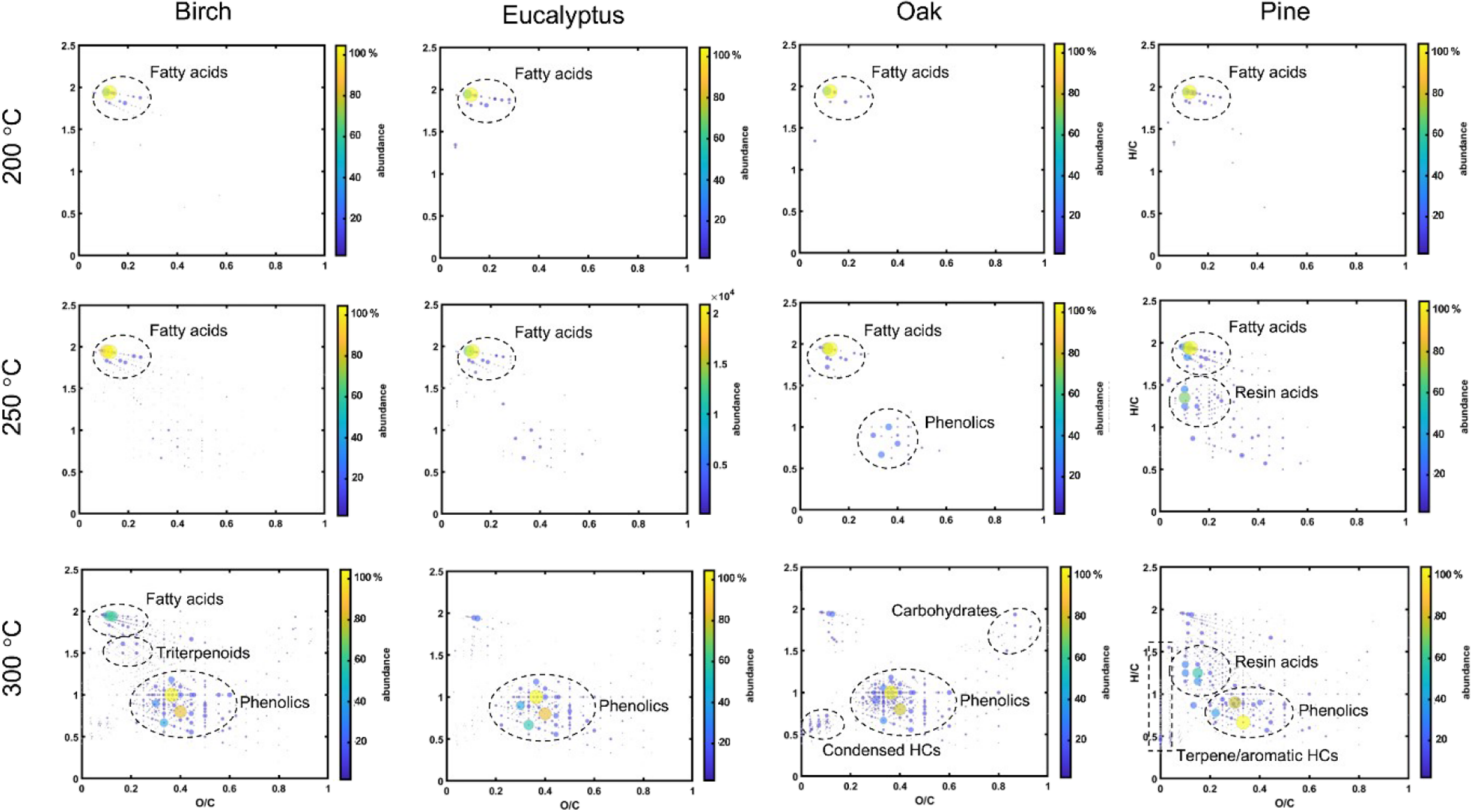
Van Krevelen diagrams for the semivolatile organic compounds (SVOCs) from model species of wood analyzed by the DIP-APCI-QTOF system. Here, pine and birch represent cases of excellent-to-good antiviral activity, whereas oak and eucalyptus represent cases of low-to-none in terms of antivirality. Similar summary of detected component groups for alder and spruce can be found in supplementary Figure S4. Key differences arise from presence of resin components in pine and rather abundant presence of phenolic compounds in both pine and birch in comparison to other tree species.

Chemical fingerprinting of SVOC compounds by DIP-APCI-QTOF mass spectrometry revealed considerable differences among wood species studied. The temperature region of 200–300 °C (desorption phase) was selected for more detailed analysis because most SVOC compounds have GC-MS analysis. Figure 7 represents the van Krevelen diagrams for the compounds detected upon thermal desorption of pine, birch, oak, and eucalyptus samples at 200 °C (2.8–3.0 min), 250 °C (4.3–4.5 min), and 300 °C (5.8–6.0 min) (for spruce and alder, see Figure S4). Thus, DIP-APCI-QTOF MS is complementary to the TD-GC-MS analysis.

The main compounds desorbed at 200 °C were saturated and nonsaturated fatty acids, showing no marked differences between the wood species. In contrast, at 250 °C pine wood showed desorption of compounds at H/C ≈ 0.1–0.3 and at O/C ≈ 1–1.5, representing different resin acids and other diterpenoids. At 250 °C, the other three wood species showed very little difference from those observed at 200 °C. The biggest differences were observed at 300 °C, where pine wood liberates a mixture of phenolic acids and aldehydes (e.g., cinnamate and coniferyl aldehyde), stilbenes and flavonoids (e.g., flavan-3-ol), and a number of resin acids. In contrast to the other wood species, pine also showed desorption of different terpene hydrocarbons, observed at O/C = 0 and ≈0.2–0.8. All hardwood species also liberated phenolic extractives at 300 °C, and oak wood also showed some condensed hydrocarbon (HC) species as well as a small number of hemicellulose-derived monosaccharides. In addition, birch wood showed the presence of triterpenoids and hydroxy/epoxy fatty acids. Spruce showed rather similar characteristics to that of pine, although considerably fewer resin acids were observed, and alder was very similar to the other hardwood species (Figure S4).

### 3.7 Modification to Natural Wood Can Significantly Alter Virus Persistence

**Figure 8:**
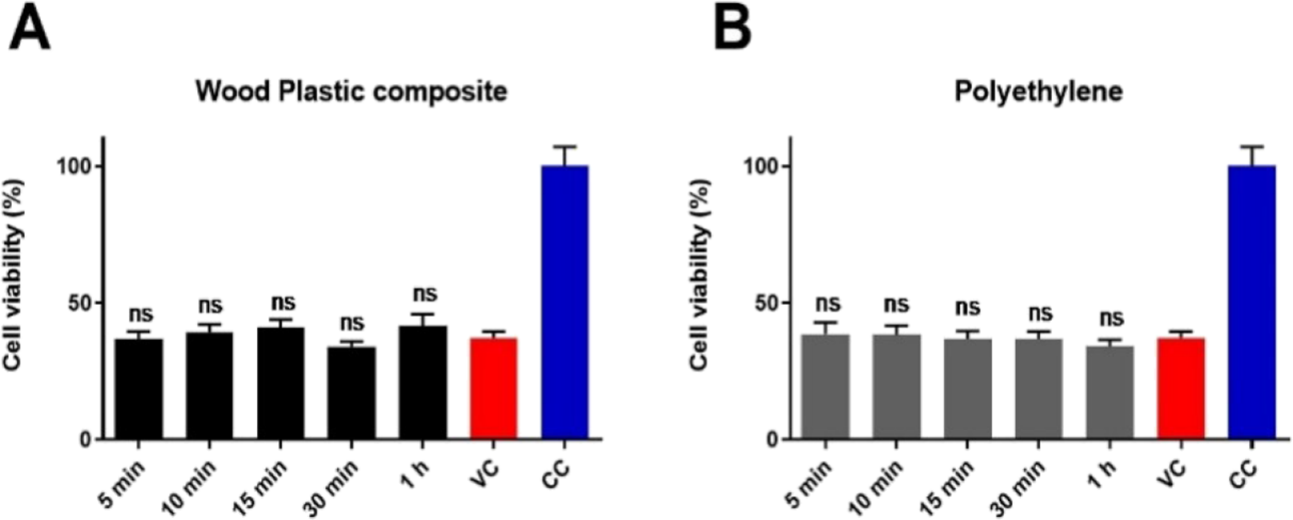
Infectivity of HCoV-OC43 recovered from (A) wood plastic composite and (B) PE plastic determined using a CPE assay. Wood composite is about 50% wood and 50% plastic, PE is used as a control plastic sample. The noninfected cell controls have been set as 100% cell viability. Results are presented as an average of four sample replicates, each including three technical replicates on MRC-5 cells. Statistical significance of the samples against virus control are shown above the bars (ns means no statistical significance).

Wood materials used in household and commercial settings are modified physically or chemically to increase their shelf life and protect them from the adverse impacts of weathering, pests, and biological degradation. To examine whether modifications such as reducing the coarseness or combining with plastic or thermal treatments would retain its antiviral efficacy against viruses, we tested the persistence of HCoV-OC43 on these modified wood surfaces. The first tests were made with a wood-plastic composite, and as a control, we used PE plastic for comparison. The HCoV-OC43 virus was added to the two surfaces and incubated for up to an hour, after which the infectivity of the viruses flushed from these surfaces from each time point was evaluated using the CPE assay. As per the CPE assay results, the viral infectivity remained unchanged even after 1 h of incubation on both these surfaces (Figure 8A, B). These results indicate that due to the presence of plastic in the wood composite, the wood composite surface acted just like the polyethylene plastic and completely lost its ability to inactivate viruses on its surface.

The effect of different coarseness levels (pine 80, 120, 320, 1000, and planed) on the persistence of HCoV-OC43 was also tested similarly. The CPE assay demonstrated that there were no differences in how the viral infectivity declined on the differently polished surfaces compared to the unpolished surface (Figures 1 and S5). For all the polished pine wood surfaces, the infectivity of the virus was lost closer to the 15 min time point. These results suggest that surface coarseness did not play a major role in altering the persistence of HCoV-OC43 on the pine wood surface.

In the third type of wood modification, we tested two types of thermally treated ThermoWood (Thermo-S and Thermo-D) pine and spruce surfaces. Thermo-S refers to indoor use product class where the dimensional stability was improved with milder temperatures (190 °C), and outdoor uses targeting Thermo-D (D stands for durability) applying a higher temperature regime (212 °C). Viruses were incubated on various surfaces for durations of 5, 10, and 15 min, and their infectivity was assessed using the CPE assay. For Thermo-S and Thermo-D pine surfaces, complete viral inactivation occurred within 15 min (Figure S6A), similar to untreated pine (Figure 1). However, Thermo-S- and Thermo-D-treated spruce showed different outcomes. While untreated spruce inactivated the virus within 10 min, Thermo-S required 15 min, and Thermo-D preserved viral infectivity throughout the incubation (Figure S6B). This implies that the thermal treatment effects vary across wood species, with Thermo-D-treated spruce potentially aiding in virus transmission. Thermo-D treatment on the spruce surface allows the viruses to persist on its surface and can be a potential fomite surface for virus transmission.

DIP-APCI-QTOF MS analysis was conducted on the thermally treated wood. The van Krevelen diagrams for SVOC compounds from Thermo-D and Thermo-S treated pine and spruce are shown in Figure S7. The results indicate that there were no marked differences between pine and either thermally treated samples. Both Thermo-S and Thermo-D samples liberated slightly higher amounts of resins at 250 and 300 °C and a lower amount of phenolics at 300 °C, as compared to the untreated wood. In contrast, different results were observed for the spruce samples. Thermo-S sample did not have marked differences from that of the untreated spruce, but Thermo-D treated spruce liberated much higher content of resin acids and phenolics as well as some carbohydrates (mono- and disaccharides), suggesting that more severe lignin and hemicellulose degradation occurs upon thermal treatment at 212 °C.

## 4 Discussion

While the antibacterial activity of wood has been extensively studied, there are only a few antiviral studies on wood materials, and especially the differences between wood species are largely unknown. Also, studies in which wood surface topography or chemical modification would have been assessed as factors contributing to antivirality are scarce. We show here that native wood materials are, in general, very good in their antiviral efficacy. Many wood species that are commonly used in indoor and outdoor housing, such as pine, spruce, and birch, killed coronavirus infectivity within a 15 min time frame. However, species like oak, eucalyptus, and alder showed much lower efficacy. Notably, enveloped and nonenveloped viruses responded differently to these surfaces.

Our CPE assay results showed that pine and spruce are the most challenging surfaces for coronaviruses to stay infectious. The infectivity was already destroyed after 5–10 min compared to alder, oak, and eucalyptus, in which viruses stayed infectious during the shortest incubation times at room temperature. It is challenging to compare these results to previous studies since only a few studies have been done with wood surfaces, and in previous studies, wood species were not described in detail. Chin et al. reported that after 2 days, no infective SARS-CoV-2 was detected on the treated wood surface in their experiments (room temperature, RH 65%) while Duan et al. reported that SARS-CoV stayed infectious for 4–5 days at room temperature on wood board.^16,23^

In our studies, we simulated standardized test conditions by incubating the virus on the surface at room temperature and over 90% RH. Additionally, it was important to note that indoor humidity levels can vary significantly, especially in Nordic countries. Wintertime conditions may see indoor humidity drop to very low levels, even below 20%, while summertime conditions typically range between 50 and 70%. In southern European countries, where temperatures can increase up to 40 °C during the summer, the environmental factors are distinctly different. To account for this variability, we conducted experiments incubating viruses on four different wood species under three different RH conditions (20, 40, and 60%) at two different temperatures (21 and 37 °C).

We observed, expectedly, that the virus persists less at higher temperatures. This has been shown by several other studies.^6,23−25^ We expected that the humidity conditions could affect the results. Humidity conditions can, for example, influence the water absorption properties of wood, which could further affect the virus’s survival on the wood surface. However, it was surprising to find that humidity played such a small role in the tested time frame. Our studies found that the relationship between RH and infectivity reduction stayed rather similar at room temperature. However, at 37 °C, we observed that moderate RH conditions destroy viral infectivity somewhat more efficiently. Thus, the relationship between RH and viral infectivity seems to be not linear, but U-shaped, as reported earlier by Casanova et al.^26^ They also suggested that the relationship between temperature and RH is different depending on the temperature conditions. It is not clear why RH would have more relevance at higher temperatures. However, we hypothesize that at higher temperatures, even subtle differences will become more pronounced. Although our results suggested a U-shaped relationship for some wood samples, such as birch and alder, it was not that for eucalyptus. The relationship therefore seems more arbitrary than repeatable.

It is known that the porosity of wood and pore diameters vary among species. This might affect the faster drying of viruses on the wood surface. Porosity and density are important parameters that significantly influence the antiviral properties of solid materials, such as flow, adsorption, and thermal conductivity. It is known that total porosity tends to decrease with increasing normal bulk density, and the morphology of the cellular microstructure of wood is more uniform for conifers (pine and spruce), and more complex for hardwoods with more versatile cell types present.^27^ Softwoods are largely composed of tracheid cells (30–50 μm across); hardwoods have smaller cells and also contain significantly larger vessel elements (50–500 μm across).

Studies applying mercury intrusion porosimetry (MIP) for measuring macro- and mesopores in the range of 58000–1.8 nm have been reported by Plötze and Niemz, Acosta et al., and Moura et al. (Table S3).^28−32^ From this setting, it is apparent that thorough analysis of the role that pore size and chemical composition of wood species have on antiviral response cannot be predicted with given sets of data alone. Modeling the relative significance of porosity and wood chemistry indicators was also initially considered to be outside the scope of this study. What is apparent, however, is the fact that wood porosity despite the species is at a scale that permits virus penetration and flushing away with relative ease. When comparing these porosity values of different wood samples to our coronavirus persistence results, it seems that the higher the porosity, the more quickly coronavirus infectivity was destroyed on the surface. We also tested the wood composite and found that virus persistence was very similar to that of plastic. These results match well with previous studies showing that viruses stay viable longer on nonporous materials than on porous materials.^33−36^ One of the explanations for faster inactivation could be the drying of the viruses. Cox reported decades ago that dehydration causes damage to the bilayer membrane of viruses and leads to other violating structural changes such as Maillard reactions of proteins and oxidation of lipids.^37^ The envelope proteins are needed in cell penetration, and thus, the damage to the viral envelope makes them inactive. Porous materials draw moisture away from absorbed viruses more efficiently, whereas, on nonporous surfaces, a moist microenvironment might enable prolonged virus survival. Chatterjee et al. reported that the bulk liquid in the respiratory droplets evaporates in minutes on both porous and nonporous surfaces.^38^ They pointed out that the critical factor is a microscopic thin residual liquid film that enables the virus to survive, despite the drying of bulk droplets. Porous materials absorb these thin films more efficiently because of the materials' fibers and pores, and thus viruses are inactivated faster. However, more specific studies supporting these physiochemical hypotheses are needed.

Our qPCR flushing experiment results indicated that wood could also retain more viral RNA than plastic. Viral particles may be physically trapped on the wood surface, which could also explain why the viral infection potential was weaker in virus samples incubated on the porous wood surfaces. The viruses might stay infectious on the surface but were stuck or absorbed inside the wood surface and did not get released to the flushing medium. This way, the number of infective viruses added to the cells was smaller and thus not so infectious for the cells in the CPE experiments. Interestingly, however, we observed that pine could not absorb enteroviruses and flushed viruses in a similar manner to the hardwood oak, which showed high antiviral efficacy despite low absorbance. It remains possible that the complex structure of coronavirus with spikes and flexible lipid envelope can be structurally more prone to adhere to wood surfaces while the very compact round nonenveloped enterovirus with very minor indentations on the surfaces is more easily released from the surface. Interestingly, as the porosity is not that different between species, e.g., pine vs oak, it would explain all loss of infectivity also in the case of coronaviruses. Therefore, the results clearly pointed to other mechanisms, i.e., antiviral compounds in the wood species themselves. However, in this study, we were not able to directly pinpoint the exact molecular details of absorption and the chemicals behind the antiviral effect. This will be the goal of future studies.

The chemical composition of wood typically includes around 40–45% cellulose, 20–25% hemicellulose, and 20–30% lignin. The rest (around 5%) of the wood composition is known as extractives. Extractives are organic compounds such as resins, flavonoids, terpenoids, essential oils, sterols, alkaloids, fatty alcohols, phenolics (such as tannins), and gums which can be extracted from the wood using polar or nonpolar solvents.^39^ While cellulose does not show any antimicrobial properties,^40^ hemicelluloses have demonstrated indirect antimicrobial activity,^41^ and lignans and extractives have repeatedly demonstrated very good antimicrobial effects.^12,42^ These extractives are known to play a major role in many functional aspects of the plant cells and improve wood’s natural resistance against decay organisms.^43^ There are some studies on the antiviral properties of extractives in the literature. For example, some diterpenoids extracted from pine are studied to inhibit viral RNA expression of influenza virus A.^44^ Tannins extracted from spruce and pine are also proven to have antiviral efficacy against nonenveloped coxsackievirus A9, as we found recently.^45^

We conducted an analysis on volatile organic components from six wood species under both dry and wet conditions at two distinct temperature settings. Notably, the species with the highest antiviral activity, namely, pine, spruce, and birch, also exhibited the most significant chemical emissions in terms of both variety and volume. While certain abundant chemicals were identified, the contribution of individual components to antiviral activity remains unspecified. Distinctly, pine and spruce contained natural resin acids and displayed a higher count of phenolic compounds and hydrocarbons compared with the other species.

Chemical analysis of spruce and Scots pine within the temperature range of 150 to 300 °C revealed an increasing presence of resin acids and phenolic compounds, characteristics of coniferous species (Figure S8). Previous studies have suggested the antiviral efficacy of lignin constituents, possibly through reactive oxygen species generated from lignin phenol oxidation.^46^ Confirming prior research by Willför et al. and Esteves et al., our findings indicated that pine emitted more resin acids, phenolic components, and certain hydrocarbons than spruce.^47−49^ However, variations were observed in the retention of some resins at higher temperatures. The prominence of phenolic compounds likely originates from lignin breakdown.

Furthermore, in thermally modified wood samples, the emergence of carbohydrates suggests the decomposition of hemicelluloses into sugar monomers.^50^ Despite a decrease in the absolute volume of chemicals in the thermally modified samples, their composition exhibited greater diversity. It appears that pine retains a higher count of antiviral resin and phenol components than spruce, potentially explaining their varied antiviral performance. The elevated presence of hemicellulose monomers in thermally modified spruce and its subsequent reduced antiviral activity might be associated with carbohydrate chain cleavage, a concept discussed by Li et al.^51^ However, the interplay between carbohydrates and viruses warrants further investigation. Our upcoming research works will assess virus viability in response to specific pure substances at defined concentrations on surfaces. Current findings, although informative, do not sufficiently pinpoint which components, lost during thermal modification, impact antiviral efficiency. The DIP-APCI-QTOF analysis (see Figure 8) demonstrates that diverse nonstructural components vaporize at varying temperatures across different wood species. Such compositional alterations can be meticulously traced, as recently shown by Castillia et al.^52^

Wood materials encountered in daily environments are often subjected to various chemical or mechanical treatments. Consequently, it is imperative to investigate the influence of these treatments on the antiviral properties of wood. Heat treatments, known to alter the physical characteristics of wood,^53^ for example, can affect the wood’s ability to absorb moisture. Our investigation of two thermally treated wood samples, pine, and spruce, revealed that pine retains its antiviral efficacy post-thermal treatment. However, in the case of spruce, increased thermal treatment was observed to diminish its antiviral efficacy, although the specific chemical mechanisms underlying this reduction remain to be clarified.

Understanding the antiviral properties of wood and the role of its chemical constituents opens the door to numerous practical applications. These findings contribute to developing antiviral surfaces, particularly in high-contact environments, structural details, and public spaces. The direct utilization of wood-based materials on surfaces with inherent antiviral properties could potentially reduce viral transmission pathways, offering a sustainable and biodegradable alternative to conventional antiviral coatings. Additionally, insights into the antiviral mechanisms of wood can inform the design of novel biobased antiviral agents based on individual chemical components or chemical mixes. This has implications not only for surface coatings but also for integrating antiviral properties into various wood products, from furniture to building materials, thereby enhancing public health safety in everyday settings.

Although we found several interesting and potentially antiviral molecules from the wood materials, it remains a limitation in this study that we could not directly pinpoint which molecules were behind the different responses of enveloped vs nonenveloped viruses. Furthermore, we could not identify the possible role of chemicals on surfaces that do not vaporize easily and, hence, were not detected by TVOC or SVOC methods. We will certainly address these questions in our future studies.

## 5 Conclusions

Altogether, our results reveal remarkable differences in the antiviral efficacy between wood species and between coronaviruses and enteroviruses. It is evident that while porosity and absorption disparities of a material contribute to its antiviral efficacy, it is primarily the chemical composition of the wood surfaces that governs the antiviral functionality. Future research will focus on identifying the most effective antiviral compounds present in wood and understanding how they interact with viruses. This could lead to innovative developments in antiviral materials inspired by these natural properties. Meanwhile, our findings also illuminate the practical utility of untreated wood surfaces as a natural effective barrier against viral transmissions, opening avenues for their application in public health strategies.

## Supporting Information Available

Detailed protocol about the immunolabeling of cells for confocal microscopy, culture, and purification of coronaviruses, methods for isolating the volatile organic compounds from wood specimens and methodology of chemical fingerprinting of semi-volatile chemicals from different wood species, tabular list of total volatile organic compounds (TVOC) emissions (top 20 selected components, total number of identified emitted chemicals) from dry and wet wood specimens and range and differences in porosity of tested tree species, photographs of experimental setup, and additional experiments supporting the results in the study (PDF)
