# Supplementary material for: Tree Species-Dependent Inactivation of Coronaviruses and Enteroviruses on Solid Wood Surfaces
Source: ACS Appl Mater Interfaces. 2024 May 28;16(23):29621–33. doi: 10.1021/acsami.4c02156 (PMC11181278; doi:10.1021/acsami.4c02156)
Supplement: Supplementary file 1 — am4c02156_si_001.pdf [file am4c02156_si_001.pdf]

Supporting information

Tree species-dependent inactivation of coronaviruses and enteroviruses on solid wood surfaces

Sailee Shroff[a#], Anni Perämäki[a#], Antti Väisänen[b], Pertti Pasanen[b], Krista Grönlund[c], Ville H. Nissinen[c], Janne Jänis[c], Antti Haapala[c,d,†], Varpu Marjomäki[a†]*

[a] Department of Biological and Environmental Sciences and Nanoscience Center, University of Jyväskylä, Jyväskylä, 40500, Finland. [b] Department of Environmental and Biological Sciences, University of Eastern Finland, Kuopio, 70210, Finland. [c] Department of Chemistry, Sustainable Technologies, University of Eastern Finland, 80100 Joensuu, Finland. [d] FSCN Research Centre, Mid Sweden University, SE-85170 Sundsvall, Sweden.

#S.S. and A.P. contributed equally to this paper

†A.H. and V.M. contributed equally to this paper

*Corresponding author

Corresponding author email address: varpu.s.marjomaki@jyu.fi

Protocol for Immunolabeling of cells

The cells were labelled with primary antibodies against the coronavirus spike protein and the cellular tubulin (#sc-58886, Santa Cruz Biotechnology) for 1 hour at room temperature. The S-protein antibody was generously provided by Moona Huttunen and Ilkka Julkunen (University of Turku). For secondary labelling, Alexa Fluor 555 goat polyclonal IgG against rabbit (catalog no. A-21429; Thermo Fisher Scientific) and Alexa Fluor 488 goat polyclonal IgG against mouse (catalog no. A-21121; Thermo Fisher Scientific) antibodies were used. Secondary antibodies were added to the cells for 50 minutes and incubated under dark conditions. Following the secondary antibody labelling, three cycles of PBS washes were given to remove any unbound secondary antibodies. The second PBS wash contained 4',6-diamidino-2-phenylindole (DAPI) (Invitrogen/Molecular Probes, ref. D3571) at a dilution of 1:40000 to stain the cellular nuclei. Imaging was conducted using a Nikon A1R confocal microscope equipped with a 40× objective and 1.25 Numerical Aperture (NA). Three lasers were employed: a 405 nm diode laser, a 488 nm multiline argon laser, and a 561 nm sapphire laser.

Coronavirus culture and purification protocol

A sub confluent layer of MRC-5 cells were cultivated in a 175 cm3 flask and infected with HCoV-OC43 (ATCC) at an MOI of 3 for 72 h, after which the supernatant was collected, and the cell debris was pelleted by centrifugation at 10,000 × g at 4℃ for 20 min. The virus in the supernatant was precipitated using 10%, wt/vol polyethylene glycol 6000 and 2.2% of NaCl. The solution was stirred for 30 min at 4℃, after which the precipitate was centrifuged at 10,000 × g at 4℃, for 30 min. The pellet was dissolved in 3 mL HEPES saline buffer [1 mM HEPES pH 6.7, 0.9% NaCl (wt/vol)] and stored on ice. The solution containing the virus appeared viscous in consistency. The virus was concentrated by pelleting through a stepwise sucrose gradient (10%–20%– 30%) at 100,000 × g at 4 °C for 2 h. The gradient was prepared by adding 3 mL of 30% sucrose to the bottom, followed by the same amount of 20% and then 10% on the top. The pellet was dissolved in 100 µL of cold HEPES saline buffer and stored at −80 °C. The infectivity of the virus batch was calculated using the end-point titration method.

Volatile organic compounds from wood specimen – method details (following chapter 2.8)

VOC emission sampling was preceded by placing the wood specimens on tinfoil plates inside the chambers (V = 114 mL) of a Micro-Chamber/Thermal Extractor M-CTE250 apparatus (Markes International Inc.) equilibrating to the target sampling temperature over 5 minutes. The VOC samples were drawn from the chambers' dedicated sampling outlets, while nitrogen served as the inert carrier gas at a 75 mL/min flow rate. Sampling lasted for 10 minutes at 25°C, and for 4 minutes at 40 °C to prevent adsorbent tube saturation.

The samples were analyzed using a thermal desorption-gas chromatography-mass spectrometry (TD-GC-MS) system consisting of a TD100 thermal desorber (Markes International Inc.) (Figure S1B), a 7890A gas chromatograph (Agilent Technologies Inc., Santa Clara, CA) equipped with an HP-5 ms UI column with 60 m length, 0.25 mm inner diameter and 0.25 µm film thickness (Agilent Technologies Inc.), and a 5975C mass spectrometer (Agilent Technologies Inc.) running on scan mode. VOC species were identified by their retention times and ion fingerprints using the MSD ChemStation software (Agilent Technologies Inc.) and NIST20 MS-library (National Institute of Standards and Technology, Gaithersburg, MD). A four-point calibration curve was constructed using HC 48 component 40,353-U

standard solution samples (Supelco Inc., Bellefonte, PA). The individual compound concentrations were calculated as toluene equivalents, according to the ISO 16000-6:2021 standard. The results were background-corrected using blank tinfoil plate samples collected concurrently with the wood specimen VOC samples.

Chemical fingerprinting of semivolatile chemicals from wood specimen – method details (following chapter 2.9)

In this work, a small amount of wood for each species (~1.5 mg) was placed inside a tip of a pre-combusted quartz capillary, sealed with a thin layer of clean quartz filter (Figure S2B). The capillary was placed inside the DIP-APCI source and vaporizer temperature was then ramped from 100 to 450 °C with 50 °C steps. After each step, the temperature was held at the target temperature for 1.5 minute before the next increment. The total heating time was therefore ~11.5 minute (Figure S3A). Three different temperature regimes can be distinguished; at 100-150 °C residual moisture and most VOC compounds are liberated (drying phase), at 200-300 °C SVOC compounds (e.g., small oxygenates, fatty/resin acids, and phenolic compounds and their derivatives) desorb (desorption phase), while at >300 °C, decomposition of hemicellulose, cellulose and lignin occurs (pyrolysis phase), which liberates a complex mixture of high-mass oxygenates and aromatic/polyaromatic hydrocarbons (HCs). The liberated compounds are then ionized in the APCI source, and their accurate masses are determined by the Q-TOF analyzer (Figure S3B, lower panel).

APCI source was operated in the negative-ion mode to obtain optimal response for oxygenated compounds. The instrument was externally mass calibrated prior to the experiments with sodium perfluoroheptanoic acid clusters within the mass (m/z) range of 50-1000. The mass spectra were further internally re-calibrated using a custom-made reference mass list (wood extractives and typical mild pyrolysis components). Following the re-calibration, extracted ion chromatograms (all ions) of the selected time regions of the desorption phase at 200, 250, and 300 °C were obtained, and elemental formulae for the compounds were obtained using the following parameters; elemental formula: 12C0-1001H0-50016O0-20; H/C ratio = 0-3; mSigma score ≤ 1000; relative abundance ≥ 0.01%; mass error ≤ 15 ppm; both odd and even electron ions allowed.

Chemical analyses – TVOC measurements using TD-GC-MS system

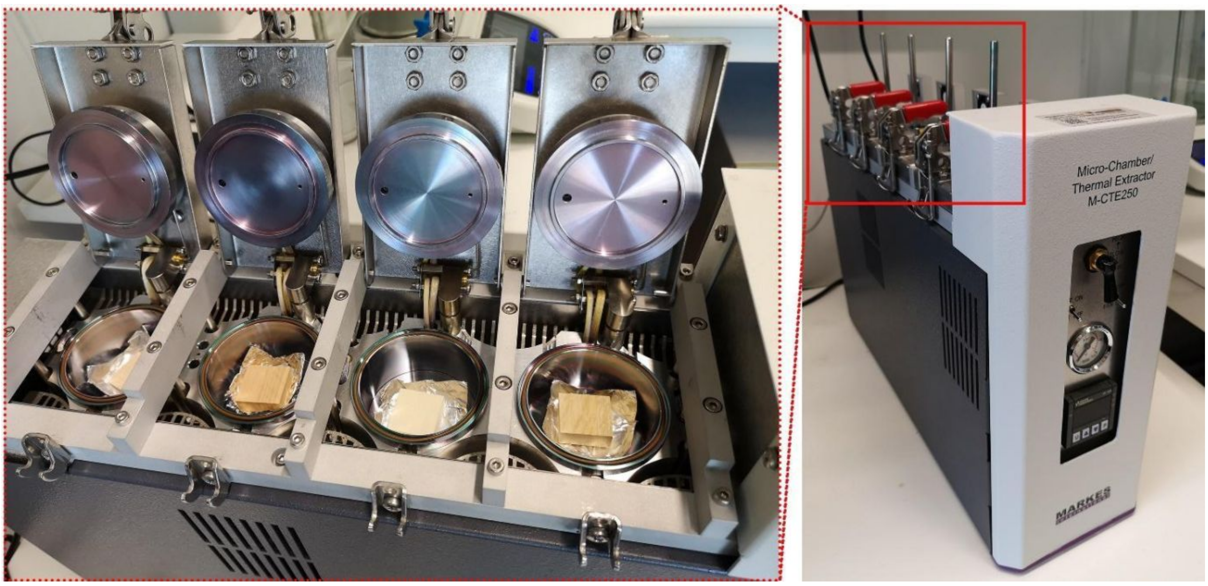

Figure S1. Measurement setup for TVOC emissions from wood specimen at 25 and 40 °C placing the wood specimens on tinfoil plates inside the chambers of a Micro-Chamber/Thermal Extractor M-CTE250 apparatus (Markes International Inc.), equilibrating to the target sampling temperature and drawing emitted VOCs via the chambers' dedicated sampling outlets.

Table S1. Total volatile organic compounds (TVOC) emissions (top 20 selected components and total number of identified emitted chemicals) from dry (moisture content ca. 8%) wood specimens in 25 °C and 40 °C incubation.

Chemical compounds emitted	Alder		Birch		Eucalyptus		Oak		Pine		Spruce	
	25 °C	40 °C	25 °C	40 °C	25 °C	40 °C	25 °C	40 °C	25 °C	40 °C	25 °C	40 °C
1-Nonene	2.1	2.6	-	-	-	-	-	-	-	-	-	-
3-Carene	-	-	-	-	-	-	-	-	-	35.3	-	-
3-Ethylpyrazoline	-	-	-	-	-	-	-	-	-	15.9	-	10.4
Acetic acid	3.8	2.6	-	47.8	30.2	44.0	-	79.8	9.7	41.2	0.5	5.5
Alpha-pinene	-	-	-	-	-	-	-	-	21.1	73.9	-	-
Benzaldehyde	-	-	-	-	-	-	-	-	-	28.5	-	-
Benzoic acid	-	1.5	-	-	-	-	-	15.9	-	9.1	-	-
Butenone	-	-	-	-	-	-	-	-	10.7	11.1	-	-
Decalactone	-	-	-	-	-	-	-	23.2	-	-	-	-
Decanal	9.7	33.1	-	5.0	22.4	42.1	-	16.7	8.5	61.9	4.0	6.8
Decanoic acid	-	-	-	-	-	-	-	33.2	-	-	-	-
Ethylhexanol	3.1	-	-	-	-	-	-	-	-	10.1	-	-
Hexanal	1.8	2.7	-	-	-	-	-	-	6.7	22.8	-	-
Linalool	3.1	3.5	-	7.7	-	-	-	-	-	-	-	-
Menthofuran	-	-	-	-	-	15.0	-	-	3.3	14.7	-	-
Nonanal	7.5	12.2	-	4.0	10.7	11.5	-	7.7	9.4	47.3	0.3	9.1
Nonene	-	-	-	-	-	15.0	-	-	-	11.5	-	-
Octanal	4.1	6.4	-	-	4.8	30.5	-	-	4.3	21.5	-	-
Hexenyl acetate	-	-	-	-	3.1	10.9	-	-	-	-	-	-
TVOC (μg/m^3/m^2)	35.2	64.5	0.0	64.5	71.3	168.9	0.0	176.5	73.7	404.9	4.8	31.8
No. of components	8	10	0.0	4	5.0	7	0.0	8	8.0	19	3.0	4

Table S2. Total volatile organic compounds (TVOC) emissions (top 20 selected components and total number of identified emitted chemicals) from briefly wetted wood specimens in 25 °C and 40 °C incubation.

Chemical compounds emitted	Alder		Birch		Eucalyptus		Oak		Pine		Spruce	
	25 °C	40 °C	25 °C	40 °C	25 °C	40 °C	25 °C	40 °C	25 °C	40 °C	25 °C	40 °C
3-Carene	-	-	-	-	-	-	-	-	934	1838	-	-
Acetic acid	66	134	271	292	40	141	446	542	-	-	207	326
Alpha-pinene	-	-	-	-	-	-	-	-	1645	3573	290	692
Alpha-terpeniol	-	-	-	-	-	-	-	-	-	-	134	321
Butanol	-	-	27	64	-	-	-	-	-	314	-	-
Decanal	14	22	-	-	18	56	9	51	-	-	-	52
Heptanal	12	20	39	83	-	-	-	-	160	283	-	72
Heptanone	-	-	33	66	-	-	-	-	107	203	-	-
Heptenone	-	-	22	50	-	-	-	-	-	-	-	-
Hexanal	437	829	656	1429	5	19	72	144	3318	7343	414	1055
Hexanoic acid	-	-	-	72	-	-	-	-	106	400	88	-
Limonene	-	-	-	-	-	-	-	-	-	-	139	279
Nonanal	15	22	24	61	10	38	9	43	120	283	-	-
Octanal	-	-	30	59	-	12	9	15	389	396	-	-
Octenal	17	28	26	72	-	-	-	-	220	314	32	58
Pentanal	51	90	81	146	-	-	5	11	758	1478	92	209
Pentanol	47	91	-	-	-	-	5	11	1045	2309	106	293
Pentenol	-	-	286	759	-	-	-	-	-	-	-	-
Pentylfuran	-	-	33	-	-	-	-	-	129	-	-	-
Styrene	42	67	68	124	-	-	35	60	-	-	-	-
TVOC (μg/m^3/m^2)	808	1566	2041	4179	97	398	615	979	10782	21816	2051	4442
No. of components	23	27	42	47	8	12	11	11	65	76	47	49

Table S3. Range and differences in porosity of tested tree species

	Normal bulk density ρ g/cm³	Porosity (MIP) %	Characteristic pore radius nm	Total specific surface m²/g
**Alder***	0.538	60.05	1062.4	74.00
**Birch***	0.594	57.12	873.5	63.01
**Eucalyptus†**	0.45	52.6	**	58.5
**Oak***	0.706	49.09	178.0	59.22
**Pine***	0.451	65.40	4044.4	121.78
**Spruce***	0.401	68.39	339.9	117.97

** data from Plötze and Niemz (2011), † Acosta et al. (2023), Kang et al. (2018), Moura et al. (2005), Alzate (2004), ** wide range of pore radii and diameter reported but results obtained with MIP method are not available.*

Chemical analyses – SVOC measurements using DIP-APCI-QTOF setup

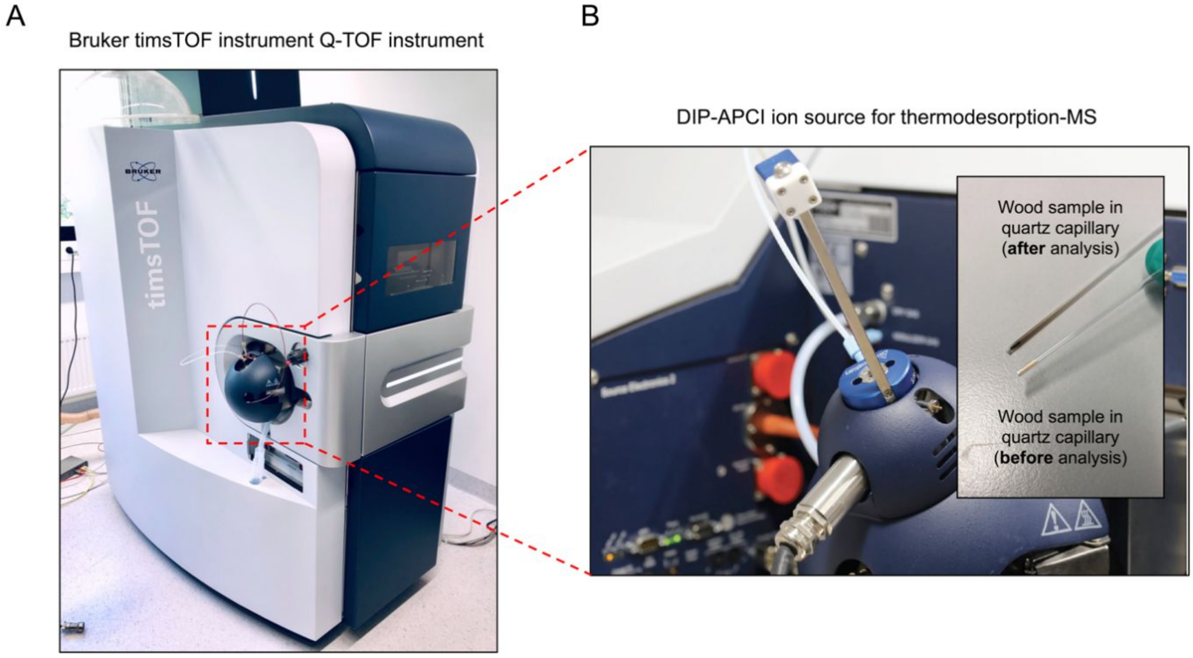

Figure S2. A) Measurement setup for DIP-APCI-QTOF chemical analysis for wood specimen and B) the outlook of wood sample in sample capillary before and after analysis (specimens were charred after measurements at 450 °C but the analyses consider only the desorption phase (see also Fig. S2).

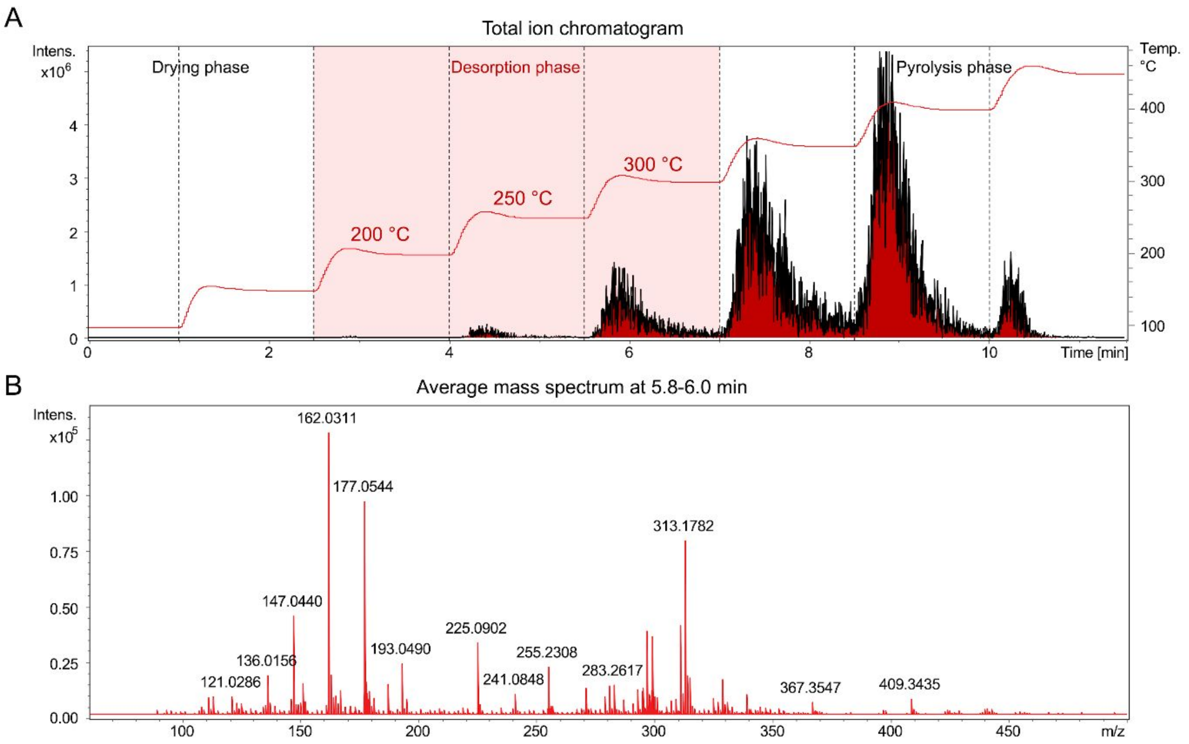

Figure S3. A) Selection of tested temperature gradient with DIP-APCI-QTOF MS and the summed abundance of detected chemical compounds in the desorption range. Chemicals observed at higher temperatures (pyrolysis phase) contain a significant number of thermal decomposition products that no longer represent the properties of native wood. Similarly, chemicals detected at below 200 °C (drying phase) were considered to have no impact on antiviral activity due to their immensely low abundance. B) Total ion chromatogram of a sample showing typical mass spectra detected while heating the sample from 250 to 300 °C which was used to identify compounds and classify them via Van Krevelen diagrams (color-coded for relative intensity) with the criteria stated in the manuscript.

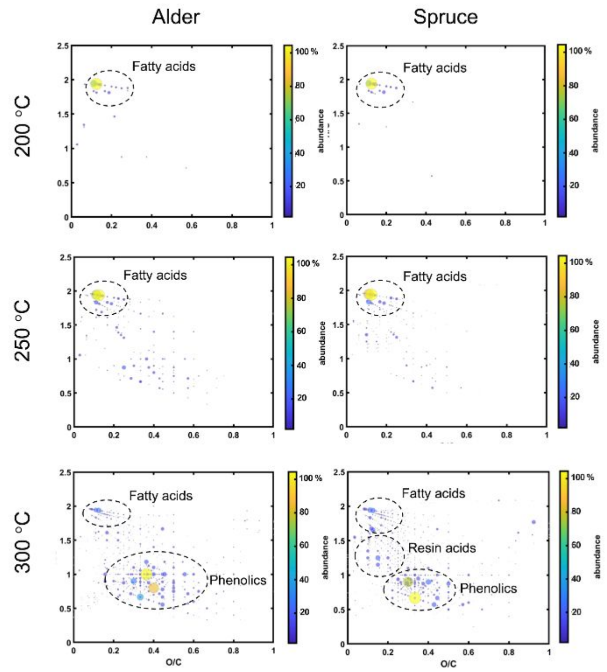

Figure S4. Van Krevelen diagrams for spruce and alder samples. The components of spruce were more numerous and closer to the (coniferous) pine wood than other tested species, including some amount of resin compounds detected at 300 °C. The components identified from alder were not significantly different from other deciduous trees like oak and eucalyptus and there were hardly any resin acid group chemicals present.

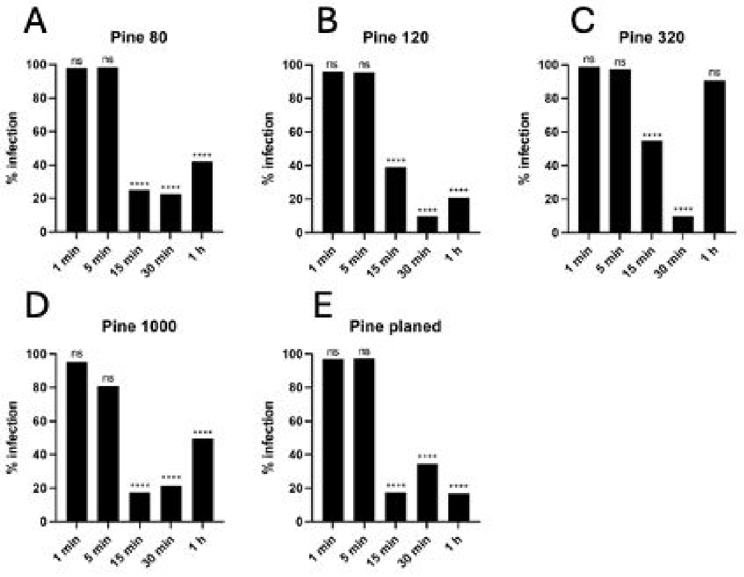

Figure S5 Infectivity of HCoV-OC43 virus recovered from different coarseness of pine (A) Pine 80, (B) Pine 120, (C) Pine 320, (D) Pine 1000 and (E) Pine planed determined using the CPE assay. The graphs have been normalized against the cell control. All the results are presented as an average of three biological replicates. Each replicate included three technical repeats on the cells. Statistical significances of the samples against virus control are shown as stars above the bars (****p < 0.0001 and ns means no statistical significance).

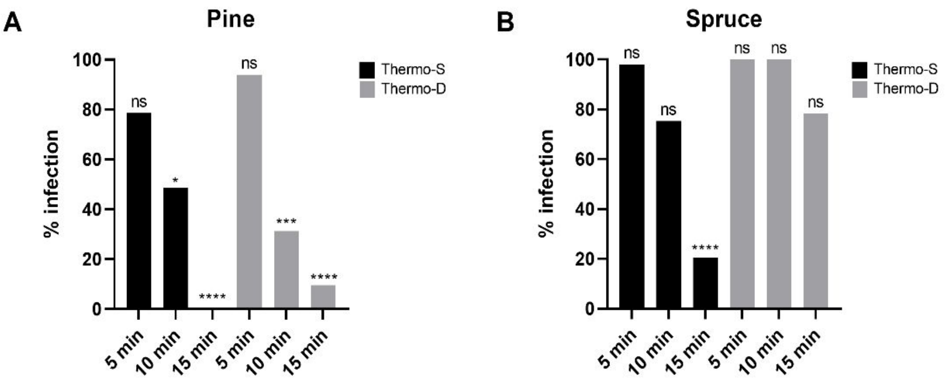

Figure S6. Infectivity of HCoV-OC43 recovered from (A) thermo-treated pine and (B) thermo-treated spruce wood samples determined using CPE assay. Thermal modifications are classified in two: Thermo-S and Thermo-D. The non-infected cell controls have been set as 100% in cell viability. Results are presented as an average of four sample replicates, each including three technical replicates on MRC-5 cells. Statistical significances of the samples against virus control are shown as stars above the bars (* $p < 0.05$, ***$p < 0.001$, ****$p < 0.0001$, and ns means no statistical significance).

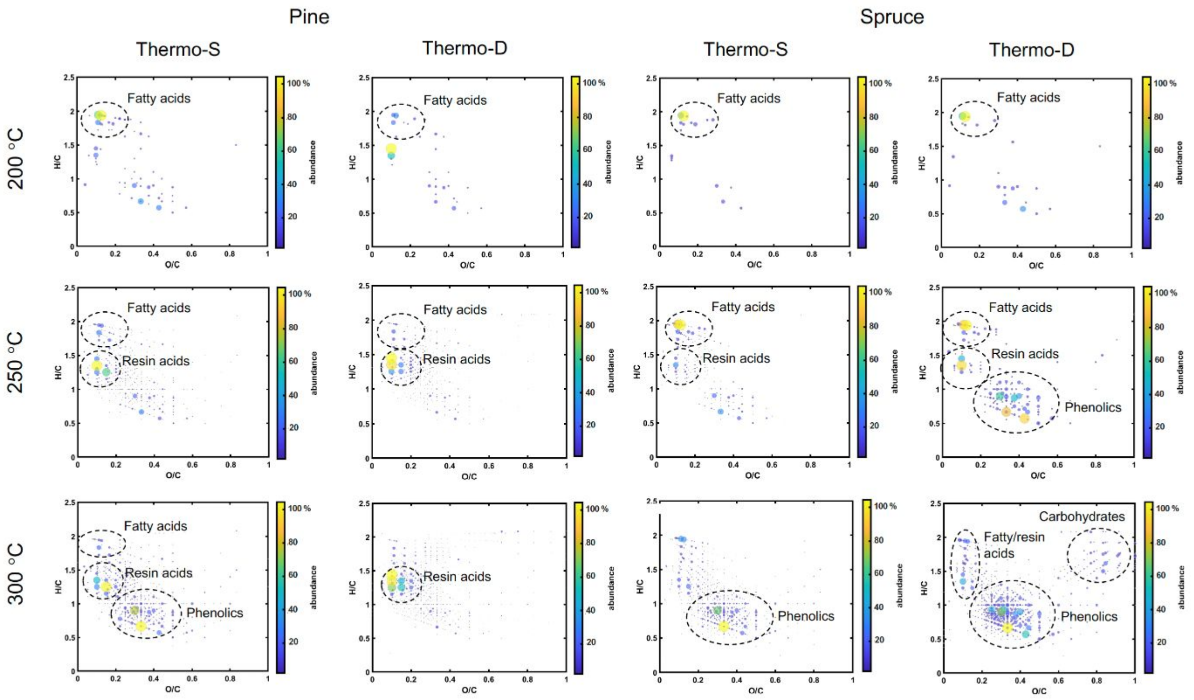

Figure S7. Van Krevelen diagrams for the compounds detected from the Thermo-S and Thermo-D treated pine and spruce samples at 200, 350, and 300 °C. Reported abundance does not directly correspond to the quantified volumes of chemical constituents. When comparing the resin, phenol and fatty acid contents of untreated spruce to Thermo-S and -D treated ones we see 2-3 times smaller quantities of chemicals emitted.

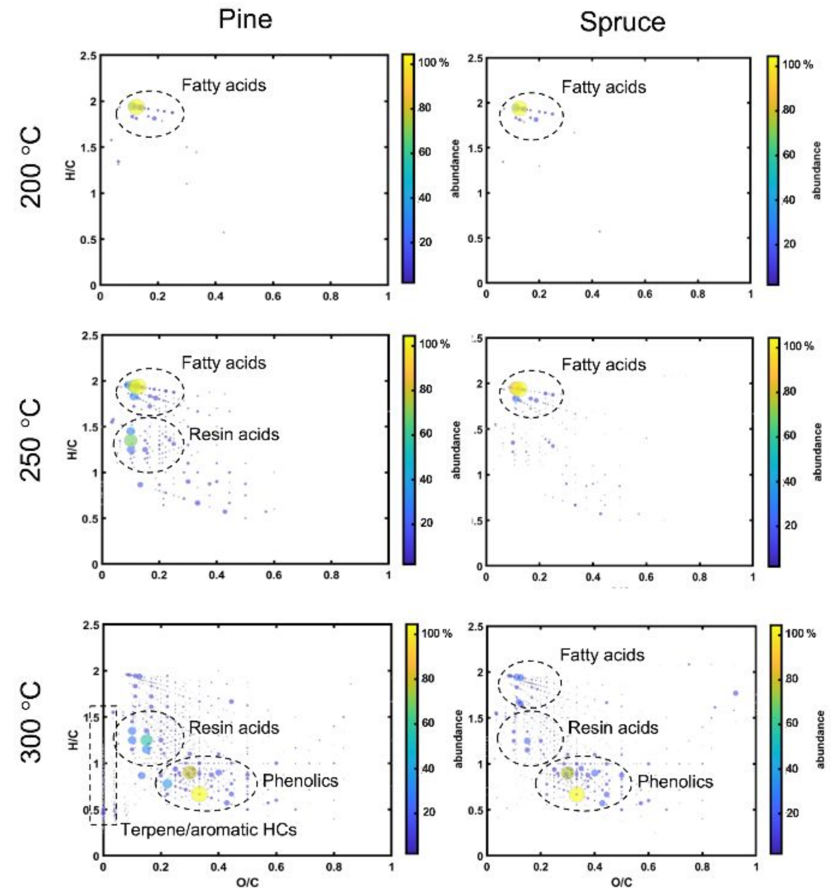

Figure S8: Van Krevelen diagrams for the semivolatile organic compound (SVOCs) from native pine and spruce analyzed by DIP-APCI-QTOF MS. Key differences arise from the presence of resin components and various aromatic hydrocarbons in pine, while smaller volume of resins arises in spruce only when measured at 300 °C. There is a rather abundant presence of phenolic compounds in both species, but their presence is more intense in pine.
